# Increased Seroprevalence and Improved Antibody Responses Following Third Primary SARS-CoV-2 Immunisation: An Update From the COV-AD Study

**DOI:** 10.3389/fimmu.2022.912571

**Published:** 2022-06-02

**Authors:** Adrian M. Shields, Sian E. Faustini, Harriet J. Hill, Saly Al-Taei, Chloe Tanner, Fiona Ashford, Sarita Workman, Fernando Moreira, Nisha Verma, Hollie Wagg, Gail Heritage, Naomi Campton, Zania Stamataki, Mark T. Drayson, Paul Klenerman, James E. D. Thaventhiran, Shuayb Elkhalifa, Sarah Goddard, Sarah Johnston, Aarnoud Huissoon, Claire Bethune, Suzanne Elcombe, David M. Lowe, Smita Y. Patel, Sinisa Savic, Alex G. Richter, Siobhan O. Burns, the COV-AD consortium, Zahra Ahmed

**Affiliations:** ^1^Clinical Immunology Service, Institute of Immunology and Immunotherapy, University of Birmingham, Birmingham, United Kingdom; ^2^Department of Clinical Immunology, University Hospitals Birmingham NHS Foundation Trust, Birmingham, United Kingdom; ^3^Institute of Immunology and Immunotherapy, University of Birmingham, Birmingham, United Kingdom; ^4^Department of Immunology, Royal Free London NHS Foundation Trust, London, United Kingdom; ^5^Institute of Translational Medicine, University of Birmingham, Birmingham, United Kingdom; ^6^Nuffield Department of Medicine, University of Oxford, Oxford, United Kingdom; ^7^Medical Research Council Toxicology Unit, University of Cambridge, Cambridge, United Kingdom; ^8^Department of Immunology, Salford Royal NHS Foundation Trust, Salford, United Kingdom; ^9^Department of Clinical Immunology, University Hospitals North Midlands, Stoke-on-Trent, United Kingdom; ^10^Department of Clinical Immunology, North Bristol NHS Trust, Bristol, United Kingdom; ^11^Department of Allergy and Clinical Immunology, University Hospitals Plymouth NHS Trust, Plymouth, United Kingdom; ^12^Department of Allergy and Clinical Immunology, Newcastle upon Tyne Hospitals NHS Foundation Trust, Newcastle, United Kingdom; ^13^Institute of Immunity and Transplantation, University College London, London, United Kingdom; ^14^National Institute for Health and Care Research (NIHR) Biomedical Research Centre (BRC) Oxford Biomedical Centre, University of Oxford, Oxford, United Kingdom; ^15^Department of Allergy and Clinical Immunology, Leeds Teaching Hospitals NHS Trust, Leeds, United Kingdom

**Keywords:** COVID-19, CVID, inborn errors of immunity, primary immunodeficiency, secondary immunodeficiency, vaccination, SARS-CoV-2

## Abstract

**Background:**

Patients with primary and secondary antibody deficiency are vulnerable to COVID-19 and demonstrate diminished responses following two-dose SARS-CoV-2 vaccine schedules. Third primary vaccinations have been deployed to enhance their humoral and cellular immunity.

**Objectives:**

To determine the immunogenicity of the third primary SARS-CoV-2 immunisation in a heterogeneous cohort of patients with antibody deficiency.

**Methods:**

Participants enrolled in the COV-AD study were sampled before and after their third vaccine dose. Serological and cellular responses were determined using ELISA, live-virus neutralisation and ELISPOT assays.

**Results:**

Following a two-dose schedule, 100% of healthy controls mounted a serological response to SARS-CoV-2 vaccination, however, 38.6% of individuals with antibody deficiency remained seronegative. A third primary SARS-CoV-2 vaccine significantly increased anti-spike glycoprotein antibody seroprevalence from 61.4% to 76.0%, the magnitude of the antibody response, its neutralising capacity and induced seroconversion in individuals who were seronegative after two vaccine doses. Vaccine-induced serological responses were broadly cross-reactive against the SARS-CoV-2 B.1.1.529 variant of concern, however, seroprevalence and antibody levels remained significantly lower than healthy controls. No differences in serological responses were observed between individuals who received AstraZeneca ChAdOx1 nCoV-19 and Pfizer BioNTech 162b2 during their initial two-dose vaccine schedule. SARS-CoV-2 infection-naive participants who had received a heterologous vaccine as a third dose were significantly more likely to have a detectable T cell response following their third vaccine dose (61.5% vs 11.1%).

**Conclusion:**

These data support the widespread use of third primary immunisations to enhance humoral immunity against SARS-CoV-2 in individuals with antibody deficiency.

## Introduction

The immunogenicity and efficacy of the initial two-dose SARS-CoV-2 vaccination schedule and booster immunisations have been comprehensively studied in healthy adults (1–4). Compared to healthy individuals, the immunogenicity of two doses of the AstraZeneca ChAdOx1 nCoV-19 and Pfizer BioNTech 162b2 SARS-CoV-2 vaccines are significantly diminished in individuals with primary (PID) and secondary immunodeficiencies (SID) (5). The COVID-19 in patients with antibody deficiency (COV-AD) study has previously shown that the seroprevalence of anti-SARS-CoV-2 antibodies is 54.8% following two vaccine doses, with seroprevalence being higher in recipients of the Pfizer vaccine, compared to the AstraZeneca (5). Other studies have estimated post-vaccine seroprevalence to lie between 20% and 80% in patients with inborn errors of immunity (6–10).

The emergence of the delta (B.1.167.2) and omicron (B.1.1.529) SARS-CoV-2 variants of concern, against which post-vaccination sera demonstrate reduced neutralising capacity (11, 12), has led to concern that the initial two-dose schedule would be insufficient to protect individuals with suboptimal vaccine responses. In September 2021, the United Kingdom Joint Committee on Vaccination and Immunisation (JCVI) recommended that individuals with significant primary or acquired immunodeficiency states receive a third primary immunisation with an mRNA based vaccine to consolidate immune responses generated from the initial two-dose immunisation schedule (13).

The immunogenicity of a third primary immunisation has been studied in certain immunocompromised cohorts; in a cohort of renal dialysis patients, seroprevalence increased from 58.9% to 98.8% following a third vaccine dose with broad cross-reactivity demonstrated against both the delta and omicron variants (14). Increases in the prevalence of neutralising antibodies have also been reported in renal dialysis patients and cancer patients following the third primary immunisation (15, 16). In a small cohort of fourteen patients with functional B cell disorders, a third mRNA vaccine dose has been shown to increase the ability of plasma samples to disrupt the interaction between the SARS-CoV-2 receptor-binding domain and the ACE2 receptor using *in vitro* binding assays as a surrogate for viral neutralisation (17). However, the immunogenicity of a third primary immunisation has not been studied in larger cohorts of individuals with PID and SID, particularly with respect to the induction of cross-reactive humoral immunity against SARS-CoV-2 variants of concern and the relative immunogenicity of heterologous versus homologous vaccine schedules.

Herein, we report the extended results of the COV-AD study, describing the waning of antibodies levels following the second vaccine dose and the response of 161 individuals with antibody deficiency to a third SARS-CoV-2 vaccine dose compared to healthy controls.

## Methods

### Patient Eligibility and Recruitment

Recruitment to the COV-AD study has been described elsewhere (5). Briefly, from March 2021, patients with primary or secondary antibody deficiency were recruited from Immunology centres across the United Kingdom. Patients were eligible for study entry if: i) they were over 18 years of age and ii) they were receiving immunoglobulin replacement therapy or they had a serum IgG concentration less than 4g/L and were receiving regular antibiotic prophylaxis to prevent infections. Participants’ underlying immunological diagnosis was made according to the European Society of Immunodeficiency Clinical Working Party criteria. In this manuscript, “other primary antibody deficiency” has been used to encompass individuals who do not fulfil the diagnostic criteria for CVID, XLA or any monogenic immunodeficiency but are still believed to have a primary humoral immunodeficiency.

Study participants were followed longitudinally through the United Kingdom routine SARS-CoV-2 vaccination schedule. Participants received two doses of either the AstraZeneca ChAdOx1 nCoV-19 (Vaxzevria) or the Pfizer BioNTech 162b2 (Tozinameran) vaccine according to the extended vaccine schedule mandated by the UK Chief Medical Officers (https://www.gov.uk/government/publications/prioritising-the-first-covid-19-vaccine-dose-jcvi-statement/optimising-the-covid-19-vaccination-programme-for-maximum-short-term-impact) between January and April 2021, followed by an mRNA third primary vaccine dose (either Pfizer BioNTech 162b2 or Moderna mRNA-1273 (Spikevax)) between September and October 2021.

Where possible, participants were sampled 1-2 months following their second vaccine dose (post V2 timepoint), up to 4 weeks prior to their third dose (pre V3 timepoint) and 1-2 months following their third vaccine dose (post V3 timepoint). When this was not possible, a single sample was taken at no fixed time point following their second vaccine dose. Participants were given the option of being sampled remotely using dried blood spots (DBS) or *via* venous blood, to enable cellular studies in addition to serology. The concordance of these methods has previously been demonstrated (18).

Prior SARS-CoV-2 infection in this study was defined as any individual who had previous PCR confirmed SARS-CoV-2 infection. In addition, any individual who demonstrated positive reactivity to pooled peptides derived from the SARS-CoV-2 nucleocapsid protein was also considered to have evidence of prior SARS-CoV-2 infection.

### Healthy Control Cohort

A cohort of 205 healthy control participants were recruited from the COVID-19 Convalescent (COCO) study. These participants were otherwise healthy health care workers, recruited from University Hospitals Birmingham NHS Foundation Trust (median age 44 years, (range 22-66 years), 28% male), vaccinated with Pfizer BioNTech 162b2 on the extended UK dosing schedule and sampled 1-2 month after vaccination. This cohort has also been followed longitudinally; 67 participants were sampled up to 1 month prior to, and 1-2 month after their third vaccine (median age 49 years (range 25-64), 28% male)

### Serological studies

A detailed account of the methods used for serological and cellular studies is available elsewhere (5). Briefly, serum or dried blood samples were tested for the presence of total anti-spike glycoprotein antibodies (The Binding Site, Birmingham, UK). Results are reported as an IgGAM ratio (optical density compared with calibrator) and results ≥1.0 are defined as seropositive. The ratio provides a semi-quantitative assessment of the magnitude of the antibody responses. Serological results are presented as the percentage of participants who are seropositive and the median of the IgGAM ratio in seropositive participants. IgG serological responses directed against the Wuhan spike protein and the B.1.1.529 (Omicron) SARS-CoV-2 variant of concern were measured using an in-house ELISA as previously described; target proteins were sourced from SinoBiological as previously described (5). Live virus neutralisation assays were performed using Vero cells on paired serum samples before and after vaccination at a 1/50 serum dilution as previously described (5).

### T Cell Studies

T cell responses were assessed using the T-SPOT^®^.*COVID* assay (Oxford Immunotec, Abingdon, UK), an ELISPOT based IFN-gamma release assay utilising peptide pools derived from the SARS-CoV-2 spike and nucleocapsid proteins; 0-4 spots per well is considered negative, 5-7 spots per cell, borderline, and greater than 7 spots per well a positive response.

### Statistical Analysis

Data were analysed using Graph Pad Prism 9.3.1 (GraphPad Software, San Diego, California USA). Continuous variables were analysed using the 2-tailed Mann-Witney U test, or the Kruskal-Wallis test with Dunn’s post-test comparison. Categorical variables analysed using the χ2 test and the relationship between antibody response, time and vaccine received by 2-way ANOVA with Tukey’s multiple comparison test.

### Ethical Approval and Funding

This study was approved by the London - Dulwich Research Ethics Committee (REC reference: 21/LO/0162) and funded by United Kingdom Research and Innovation (MR/W002663/1). Serological responses from healthy individuals are from participants recruited to the COVID-19 Convalescent (COCO) immunity study (REC reference 20/HRA/1817). All participants provided written informed consent prior to participation in this study.

## Results

We present analysis of the response to SARS-CoV-2 vaccination of 182 COV-AD participants sampled up to 2 months following their second vaccine dose (post V2 timepoint - median time from second vaccination: 45 days), 111 participants sampled 1 month before their third primary immunisation (pre V3 timepoint - median: 174 days from second vaccine, 17 days before third vaccine) and 161 participants sampled up to 3 months after their third primary immunisation (post V3 timepoint - median: 47 days from third vaccine). Demographic information for these groups are provided in **Table 1**. The overwhelming majority of participants (88.8-93.4%) in each group were receiving immunoglobulin replacement therapy for antibody deficiency. Between 59.3% and 63.3% of participants in each group received the AstraZeneca ChAdOx1 nCoV-19 vaccine for their initial two doses. 97.7% of participants received an mRNA-based third primary immunisation, 94.4% of them the Pfizer BioNTech 162b2 vaccine.

**Table 1:** Demographics of COV-AD study participants.

	Post V2	Pre V3	Post V3
**Participants (n)**	182	111	161
**Age (yr, IQR)**	59 (42-69)	64 (46-72)	63 (51-71)
**Sex (n, % Male)**	72 (39.6)	46 (41.4)	69 (42.8)
**Prior PCR+ infection (n, %)**	11 (6.0)	10 (9.0)	11 (6.8)
**Initial vaccination (n, %)**			
*AstraZeneca CHADOX1 nCOV-19*	108 (59.3)	63 (56.8)	102 (63.3)
*Pfizer BNT162b2*	72 (39.6)	48 (43.2)	59 (36.6)
*Unknown*	2 (1.1)	0 (0.0)	0 (0.0)
**Third primary vaccination (n, %)**			
*AstraZeneca CHADOX1 nCOV-19*	-	-	2 (1.2)
*Moderna mRNA-1273*	-	-	7 (4.3)
*Pfizer BNT162b2*	-	-	152 (94.4)
*Unknown*	-	-	1 (0.6)
**Median sampling time (d)**	45.0 (Post V2)	174.0(Post V2) 17.0 (Pre V3)	47.0 (Post V3)
**Immunoglobulin replacement (n, %)**			
*IVIG*	105 (57.7)	51 (45.9)	62 (38.5)
*SCIG*	65 (35.7)	48 (43.2)	81 (50.3)
*Antibiotic prophylaxis only*	11 (6.0)	10 (9.1)	12 (7.4)
*Unknown*	1 (0.5)	2 (1.8)	6 (3.7)
**Pre-treatment IgG (g/L)**	3.5	3.7	3.2
**Diagnoses**			
**Primary immunodeficiency (n,%)**	117 (64.3)	81 (73.0)	107 (67.1)
Common variable immunodeficiency	73	47	69
Primary antibody deficiency	14	18	15
Specific polysaccharide antibody deficiency	7	7	8
X-linked agammaglobulinaemia	7	4	3
X-linked hyper IgM syndrome	4	1	1
GATA2 immunodeficiency	0	1	0
Goods syndrome	1	1	2
Undefined combined immunodeficiency	4	1	5
APDS1	1	1	1
Autoimmunelymphoproliferative syndrome	0	0	0
CTLA-4 haploinsufficiency	2	0	0
STAT1 gain of function	1	0	0
NFKB2 haploinsufficiency	1	0	0
SAMD9L loss of function	1	0	0
X-linked SCID post gene therapy	1	0	0
**Secondary immunodeficiency (n,%)**	65 (35.7)	30 (27.0)	53 (32.9)
Other/not specified	0	0	2

**Figure 1:**
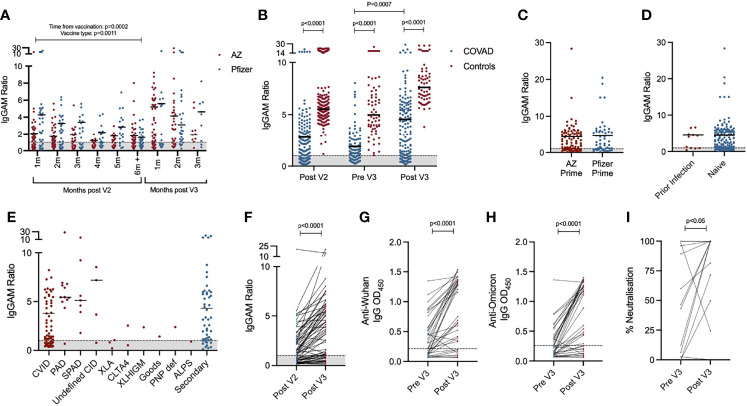
Serological response to third primary immunisation in COV-AD participants: **(A)** Total anti-SARS-CoV-2 spike glycoprotein antibodies in all participants sampled throughout the COV-AD study presented by time of sampling relative to V2 and V3. **(B)** Comparison of total anti-SARS-CoV-2 spike serological responses of infection naive COV-AD participants and healthy controls 1-2 months post second vaccine dose (Post V2), up to 1 month prior to third vaccine dose (Pre V3) and 1-2 months post third vaccine dose (Post V3). **(C)** Comparison of total anti-SARS-CoV-2 spike serological responses of infection naive COV-AD participants 1-2 months post third vaccine dose by initial two-dose vaccine received. **(D)** Comparison of total anti-spike serological responses of COV-AD participants 1-2 months post third vaccine dose by prior infection status. **(E)** Comparison of total anti-spike serological responses of infection naive COV-AD participants 1-2 most post third vaccine dose by underlying immunodeficiency. **(F)** Comparison of total anti-spike antibody levels of paired samples taken 1-2 months after the second vaccine dose and 1-2 months after the third vaccine dose. **(G)** Comparison of IgG binding to the original Wuhan SARS-CoV-2 spike glycoprotein between paired samples taken before and after third primary immunisation. **(H)** Comparison of IgG binding to the B.1.1.529 (Omicron) SARS-CoV-2 spike glycoprotein between paired samples taken before and after third primary immunisation. **(I)** Neutralisation capacity of paired serum samples taken before and after third vaccine dose against SARS-CoV-2 in a live virus neutralisation assay. In all cases, the grey shaded area represents the assay cutoff.

In the six month period following the second vaccine dose, anti-SARS-CoV-2 spike antibody levels, but not seropositivity, significantly decreased (2-way ANOVA, p=0.0002) (**Figure 1A**). A third primary immunisation significantly increased seropositivity in the COV-AD cohort (Seropositive %: Pre V3 - 61.4% vs. Post V3 - 76.0%, Chi Square 6.15, p=0.013) but the percentage of participants with detectable antibody responses remained lower than healthy controls, all of whom were seropositive, at all time points (Chi square: COV-AD vs healthy controls at Post V2, Pre V3 and Post V3; p<0.0001). A third vaccine dose also increased the magnitude of the antibody responses in COV-AD participants (2-Way ANOVA; Tukey’s multiple comparison test - IgGAM ratio: Pre V3 1.88 vs. Post V3 4.54, p=0.0007). However, in comparison to healthy controls, these responses were significantly lower at every sampling point (2-way ANOVA, p<0.0001) (**Figure 1B**; **Table 2**). A third vaccine dose increased seroprevalence and humoral responses in all major disease subgroups: CVID, primary antibody deficiency, SPAD and secondary immunodeficiency (**Table 2**; **Supplementary Figure 1**). Immunological correlates of post-vaccine seropositivity following the third vaccine dose were similar to those we have previously reported (5): vaccine responders had higher pre-treatment IgG level (mean IgG 3.7g/L vs. 2.02g/L, p<0.0001) and larger populations of peripheral blood CD19+ B cells (mean CD19 population 0.50x109/L vs 0.12x109/L, p=0.042). There were no significant differences in trough IgG concentrations between seropositive and seronegative participants receiving immunoglobulin replacement (9.75g/L vs 9.25g/L, p=0.276) and no significant differences in trough IgG concentrations amongst vaccine responders when these participants were analysed by quartiles, based on their level of anti-SARS-CoV-2 spike antibodies post V3 (Kruskal-Wallis statistic 7.38, p=0.12). It is therefore unlikely that any antibodies potentially present in immunoglobulin products significantly contribute to the serological responses observed during this study.

**Table 2:** Summary of serological response to third primary immunisation in infection-naive COV-AD participants.

Timepoint	Post V2 (n=171)			Pre V3 (n=101)			Post V3 (n=150)		
	N	Sero-positivity (%)	Median IgGAM	N	Sero-positivity (%)	Median IgGAM	N	Sero-positivity (%)	Median IgGAM
Healthy controls	205	100%	5.51	68	100%	4.97	68	100%	7.61
All COVAD participants	171	55.6%	2.81	101	61.4%	1.88	150	76.0%	4.54
CVID	70	52.9%	2.81	43	60.5%	1.58	62	66.1%	3.79
PAD	12	75.0%	2.45	15	53.3%	2.47	13	92.3%	5.45
SPAD	6	100%	2.71	7	100%	1.89	7	100%	5.11
Secondary	62	59.6%	3.03	28	64.3%	2.37	51	82.3%	4.30

Two doses of the Pfizer BioNTech 162b2 vaccine has previously demonstrated superior humoral immunogenicity in individuals with antibody deficiency compared to two doses of AstraZeneca ChAdOx1 nCoV-19 (5). In the 6 months following V2, this superiority was maintained, but the effect lessened over time (2-way ANOVA; p=0.0011) (**Figure 1A**). At 6 months post V2, there was no significant difference in seroprevalence (AZ 56.1% vs. Pfizer 55.6%, NS) or the magnitude of the antibody response (IgGAM ratio: AZ 1.8 vs. Pfizer 1.58, NS) between recipients of the two vaccines. Furthermore, following the third primary immunisation, there was no significant difference in the percentage of seropositive individuals or the magnitude of the antibody response amongst individuals who initially received two doses of AstraZeneca and those who received two doses of Pfizer (% Seropositive: AZ 76.8% vs. Pfizer 73.6%, NS; IgGAM ratio: AZ 4.35 vs. Pfizer 4.63, NS) (**Figure 1C**). Prior PCR proven SARS-CoV-2 infection also did not significantly increase the magnitude of the serological response, although the numbers of patients with prior infection was low (**Figure 1D**).

Heterogeneity in the antibody response to vaccination was observed amongst individuals with primary and secondary immunodeficiencies (**Figure 1E**). Individuals with X-linked agammaglobulinaemia (XLA) did not mount an antibody response to vaccination, in keeping with the molecular mechanism underlying their immunodeficiency. Amongst the other major disease groups, individuals with CVID demonstrated the lowest post-third vaccine dose seroprevalence and lowest median antibody response following the third vaccine dose. In contrast, individuals with SPAD, who by definition have not responded to prior pneumococcal vaccination, all responded to COVID vaccination. (**Table 2**; **Supplementary Figure 1**).

Paired samples were available from 64 participants bled 1-2 months after their second dose and 1-2 months after their third dose (**Figure 1F**). Overall seroprevalence in these participants rose from 59.4% after V2, to 75.0% after V3 demonstrating the immunogenicity of a third primary vaccination in non-responders to the first two doses. Paired samples from the same participant were available from 37 participants before and after the third vaccine dose. Using ELISAs, these samples were analysed for levels of IgG directed against the original Wuhan SARS-CoV-2 spike protein and the B.1.1.529 SARS-CoV-2 variant of concern (Omicron) (**Figures 1G, H**). Seroprevalence of IgG antibodies directed against the Wuhan and Omicron spike proteins was 62.2% and 40.5% respectively prior to the third vaccine dose. Following third primary immunisation, the seroprevalence of antibodies directed against the Wuhan spike protein increased to 78.4% (Chi square 1.62, not significant) and significantly increased to 67.5% against the Omicron spike protein (Chi square 4.44, p=0.02), demonstrating the ability of a third primary immunisation to induce cross-reactive antibodies directed against dominant SARS-CoV-2 variant of concern in early 2022. However, seroprevalence against the Omicron variant amongst COV-AD participant remains significantly lower than healthy controls (COV-AD vs healthy controls: 67.5% vs 100%, Chi Square 22.2, p<0.0001). The functionality of the antibody response was investigated using live virus neutralisation assays directed against the Wuhan SARS-CoV-2 strain: third primary immunisation was associated with a significant increase in serum neutralisation capacity (median % neutralisation: Pre V3 44.8% vs. Post V3 99.8%, p=0.0479, n=14 paired serum samples) (**Figure 1I**).

**Figure 2:**
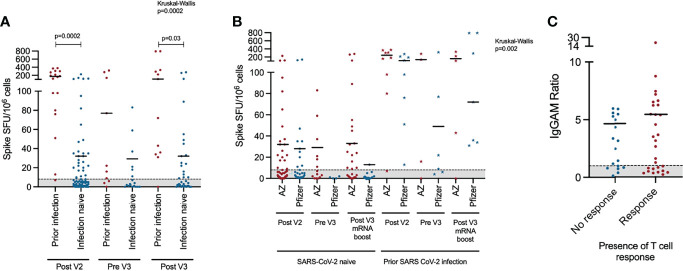
T cell response to third primary immunisation in COV-AD participants: **(A)** Longitudinal comparison of T cell responses measured using the T-SPOT^®^.*COVID* assay in COV-AD participants by prior infection status. **(B)** Longitudinal comparison of T cell responses measured using an interferon-gamma release assay in COV-AD participants by initial two-dose vaccine schedule received. Data points represented by stars are individuals with evidence of prior infection. In both cases, the grey shaded area represents the assay cutoff. **(C)** Comparison of IgGAM ratios of individuals with detectable and undetectable T cell responses.

T cell responses to infection and vaccination were measured by ELISPOT **(** **Figure 2**). Overall, T cell responses were detectable in 47.5% (n=47/95) of individuals after the second vaccine dose, 46.4% of individuals prior to their third vaccine dose (n=13/28) and 59.6% (n=28/47) of individuals after their third vaccine dose. Individuals with prior PCR-proven SARS-CoV-2 infection were more likely to have a detectable T cell response (Post V2: 94.1% vs 38.8%, Pre V3: 70.0% vs 33.3%, Post V3: 91.6% vs 48.6% - Chi Square p<0.05 Post V2 and Post V3) and that T cell response was quantitatively greater (Kruskal-Wallis 24.5, p=0.002; Dunn’s multiple comparison tests: Post V2 prior infection vs infection naive: p=0.0002. Post V3 prior infection vs infection naive: p=0.03) (**Figure 2A**). T cell responses were more frequently detectable in individuals with prior infection regardless of their vaccine schedule (**Figure 2B**). However, in SARS-CoV-2 infection-naive individuals, a greater percentage had a detectable T cell response at the V3 timepoint if they had received a heterologous third vaccine dose (i.e. two doses of ChAdOx1 nCoV-19 followed by an mRNA vaccine) compared to those who had received three consecutive doses of mRNA vaccines (heterologous vs homologous: 61.5% vs 11.1%, Chi square 6.81, p=0.009). There was no statistically significant relationship between the magnitude of the antibody response to vaccination and the presence or absence of a detectable T cell response (**Figure 2C**).

In the subgroup of participants with both antibody and T cell results after V3 (n=47), only 10.6% (n=5/47) failed to make either an antibody response or a T cell response. There were no common demographic or clinical characteristics shared between these non-responders: three individuals had CVID (2 with bronchiectasis, 1 with GL-ILD), one had XLA and one secondary antibody deficiency following cardiac transplantation. The age of these participants ranged from 34 to 84 years; 80% were male. Three participants received two doses of the AZ vaccine followed by a third dose of Pfizer, the others received three doses of the Pfizer vaccines. All had CD4+ T cell count within the normal range and 60% had a normal B cell count. The only participant receiving immunosuppression medication was the individual with secondary immune deficiency (daily corticosteroids and tacrolimus).

## Discussion

Vaccination remains the most effective intervention to prevent SARS-CoV-2 morbidity and mortality in the general population. To date, the most robust correlate of protection against SARS-CoV-2 infection and severe COVID-19 is the presence of anti-viral neutralising antibodies (19, 20). We have previously demonstrated that individuals with primary and secondary antibody deficiency demonstrate reduced seroprevalence of anti-spike antibodies following the initial two-dose vaccination schedule and, compared to healthy controls, reduced magnitude of antibody responses and reduced *in vitro* neutralising capacity (5). Through longitudinal monitoring we demonstrate significant waning of the magnitude of the antibody response following a two-dose vaccine schedule and the immunogenicity of a third vaccine dose.

Following third primary immunisation with an mRNA-based vaccine, the overall seroprevalence of anti-spike glycoprotein antibodies in the COV-AD cohort rose from 61.4% immediately before, to 76.0% after vaccination and was accompanied by a significant increase in the median antibody levels amongst vaccine responders. The serological response induced by the third primary immunisation was broadly cross-reactive against the omicron SARS-CoV-2 variant of concern and the neutralisation capacity of paired serum samples also increased, an important observation given neutralising antibodies are associated with protection against SARS-CoV-2 infection and severe disease (20). These data are concordant with similar studies in renal dialysis (14, 15) and cancer patients (16) and small studies of individuals with functional B cell defects (17). Nevertheless, seroprevalence and the magnitude of antibody responses in this cohort remain significantly lower than healthy controls. Even after the third vaccine dose, 32.5% of individuals in this study failed to demonstrate antibody binding to the currently dominant Omicron variant.

Our study highlights the heterogeneity of vaccine responsiveness in primary and secondary antibody deficiencies. A third of individuals with CVID demonstrate no humoral response to SARS-CoV-2 vaccination, and the magnitude of the response is variable amongst responders. In contrast, all individuals with SPAD were SARS-CoV-2 vaccine responsiveness, albeit at lower levels that healthy controls, despite having failed to respond to prior pneumococcal vaccination. Given the rapid emergence of novel SARS-CoV-2 variants, it will be important to understand the prevalence and clinical characteristics of individuals who are intrinsically vaccine unresponsiveness and those whose immunity may be improved by heterologous or variant-specific vaccination strategies. Understanding the molecular mechanisms governing this heterogeneity may also allow stratification of the severity of immune deficiency and inform future vaccine design.

Heterologous vaccination strategies have been associated with greater serological responses following a third vaccine dose in healthy control cohorts (1, 21), In contrast, we did not observe a significant difference in the serological responses to a third dose of mRNA vaccine based upon whether an individual initially received two doses of the adenovirally-vectored ChAdOx1 nCov-19 or the mRNA Pfizer BioNTech 162b2 vaccines. This is concordant with a study of cancer patients, where neutralising antibody titres after a third vaccine dose were not affected by the initial vaccine schedule in multiple logistic regression analysis (16). However, in infection naive individuals, a heterologous vaccination strategy was associated with a significantly greater percentage of individuals having a detectable T cell response. These results should be interpreted cautiously; an ELISPOT assay only describes one aspect of the T cell response to infection and/or vaccination. However, previous studies have shown superior spike-specific T cell responses in the over-80s following the ChAdOx1 nCov-19 vaccine (22), more durable preservation of spike-specific CD8+ following the adenovirally vectored Ad26.COV2.S (Johnson and Johnson) immunisation (23) and induction of superior T cell responses using heterologous third dose vaccine schedules (24). Although, the relationship between T cell responses to vaccination and protection from SARS-CoV-2 infection and severe disease remain uncertain, these findings may be relevant to individuals incapable of making any humoral immune responses, for example those with XLA.

It remains uncertain whether individuals who have failed to mount a serological response to vaccination after three vaccine doses would benefit from further immunisations, and, if so, with what vaccine. In the absence of established serological thresholds that correlate with protection against infection and severe disease, particularly against novel variants of concern, it also remains uncertain whether the wider antibody deficient cohort would also benefit from further vaccinations to enhance antibody titres and seropositivity. In this cohort, humoral vaccine non-responsiveness was associated with lower peripheral B cell numbers and lower pre-treatment IgG concentrations. Yet, we and others have documented survival and SARS-CoV-2 viral clearance in the absence of either B cells or detectable humoral immunity (5, 25), suggesting SARS-CoV-2 neutralising antibodies are but one aspect of protective immunity against the virus. Longitudinal vaccine efficacy studies in individuals with immunodeficiency are necessary to develop a comprehensive understanding of the relationship between measurable *in vitro* immunological parameters and protection against severe disease, and allow further stratification of the ongoing risk posed by COVID-19 (26, 27).

In the absence of this understanding, we recommend ongoing caution with respect to the risk of SARS-CoV-2 in individuals with PID and SID, particularly amongst those who have no detectable immune response to vaccination. Such individuals should be prioritised for urgent access to clinically proven antiviral treatments (28) and/or monoclonal antibodies (29) in the event of testing positive for SARS-CoV-2, and where available, pre-exposure prophylaxis with monoclonal antibodies (30). Given the waning following the 2nd dose of vaccination it is important to understand whether the kinetics of the antibody waning will be similar after the 3rd dose of vaccination and the booster 4th dose due to be given in the UK in the spring of 2022. This will be essential to understanding how regularly we should be vaccinating this cohort going forward.

In conclusion, we present evidence that, in a cohort of patients with antibody deficiency, a third primary SARS-CoV-2 immunisation is associated with increased seroprevalence and antibody levels, the induction of cross-reactive antibodies against SARS-CoV-2 variants of concern and enhanced neutralisation capacity against the SARS-CoV-2 virus. Although vaccine responsiveness remains significantly lower than healthy controls, these data strongly support the widespread use of a third primary immunisation in immunodeficient patients.

## Data Availability Statement

The raw data supporting the conclusions of this article will be made available by the authors, without undue reservation.

## Ethics Statement

The COV-AD study was approved by the London - Dulwich Research Ethics Committee (REC reference: 21/LO/0162). Serological responses from healthy individuals are from participants recruited to the COVID-19 Convalescent (COCO) immunity study (REC reference 20/HRA/1817). The patients/participants provided their written informed consent to participate in this study.

## COV-AD Consortium

Zahra Ahmed, Angus Best, Joanne Dasgin, Mohammad Dinally, Elena Efstathiou, Theresa McCarthy, Madeeha Hoque, Shannon Page, Timothy Plant, Zehra Suleiman, Neil Townsend, Charlotte Trinham, Sinead Walder, Clinical Immunology Service, Institute for Immunology and Immunotherapy, University of Birmingham, UK; Hollie Bancroft, Michelle Bates, Hayley Clifford, Christopher McGee, University Hospitals Birmingham NHS Foundation Trust, Birmingham, UK; Samuel Chee, Lucy Common, Archana Herwadkar, Karen Knowles, Maria Poulaka, Department of Immunology and Allergy, University Hospital Plymouth NHS Trust, Plymouth, UK; Georgina Davis, Daniel Mullan, Stuart Wareham, Department of Immunology, Salford Royal NHS Foundation Trust. Salford, UK; Fatima Dhalla, Rashmi Jain, Hadeil Morsi, Nicholas Peters, Department of Clinical Immunology, Oxford University Hospitals NHS Foundation Trust, Oxford, UK; Mark Gompels, Malgorzata Slowinsksa, Department of Immunology, North Bristol NHS Trust, Bristol, UK; Dan Hartland, Saving Lives Charity, MIDRU Building, Heartlands Hospital, Birmingham, UK; Emily Heritage, Joe Humphreys, Institute of Translational Medicine, University of Birmingham, Birmingham, UK; Deborah Hughes, Ann Ivory, Department of Immunology, University Hospital North Midlands, Stoke, UK; Sinead Kelly, Newcastle Upon Tyne Hospitals NHS Foundation Trust, Newcastle, UK; Eileen O’Grady, Department of Allergy and Clinical Immunology, Leeds Teaching Hospitals NHS Trust, Leeds, UK; Archana Shajidevadas, Research and Development Department, University Hospital Plymouth NHS Trust, Plymouth, UK

## Author Contributions

AS, SB, and AR designed and supervised the study. SF, HH, SA-T, CT, FA, and ZS undertook experimental work and analysis for the study. MD supported the development of the ELISAs used to investigate serological responses to novel variants of concern. SW, FM, NV, SG, SJ, AH, CB, SuE, DL, SP, SS, AS, SB, and AR recruited patients to the study and acted as local site principal investigators. HW, GH, and NC provided administrative and database support for the study and facilitated patient recruitment to the study. JT, PK, SB, and AR provided senior leadership and strategic oversight for the study. AS analysed the data, wrote the first draft of the manuscript and revised the manuscript. All authors contributed the revision of the manuscript and read and approved the final version.

## Funding

This study was funded by United Kingdom Research and Innovation (MR/W002663/1). The COCO study, provided healthy control participants and was carried out at the National Institute for Health Research (NIHR)/Wellcome Trust Birmingham Clinical Research Facility (BRC-1215-20009). The views expressed are those of the authors(s) and not necessarily those of the NHS, the NIHR or the Department of Health.

## Conflict of Interest

The authors declare that the research was conducted in the absence of any commercial or financial relationships that could be construed as a potential conflict of interest.

## Publisher’s Note

All claims expressed in this article are solely those of the authors and do not necessarily represent those of their affiliated organizations, or those of the publisher, the editors and the reviewers. Any product that may be evaluated in this article, or claim that may be made by its manufacturer, is not guaranteed or endorsed by the publisher.

## Abbreviations

ACE2, Angiotensin converting enzyme 2; APDS-1, Activated PI3K delta syndrome 1; CID, Combined immunodeficiency; COV-AD, Coronavirus in patients with antibody deficiency; COVID-19, Coronavirus disease 2019; CTLA-4, Cytotoxic T-lymphocyte-associated protein 4; CVID, Common variable immunodeficiency disorder; DBS, Dried blood spot; GATA2, GATA-binding factor 2; ELISA, Enzyme-linked immunosorbent assay; JCVI, Joint Committee on Vaccination and Immunisation; NFKB2, Nuclear Factor Kappa B Subunit 2; PAD, Primary antibody deficiency; PCR, Polymerase chain reaction; SAMD9L, Sterile Alpha Motif Domain Containing 9 Like; SARS-CoV-2, Severe acute respiratory syndrome coronavirus 2; SID, Secondary immunodeficiency; SPAD, Specific polysaccharide antibody deficiency; STAT1, Signal transducer and activator of transcription 1; V2, Vaccine 2 (timepoint); V3, Vaccine 3 (timepoint); XLA, X-linked agammaglobulinaemia.
