# Supplementary material for: Increased Seroprevalence and Improved Antibody Responses Following Third Primary SARS-CoV-2 Immunisation: An Update From the COV-AD Study
Source: Front Immunol. 2022 Jun 2;13:912571. doi: 10.3389/fimmu.2022.912571 (PMC9201027; doi:10.3389/fimmu.2022.912571)
Supplement: Supplementary file 1 [file Presentation_1.pdf]

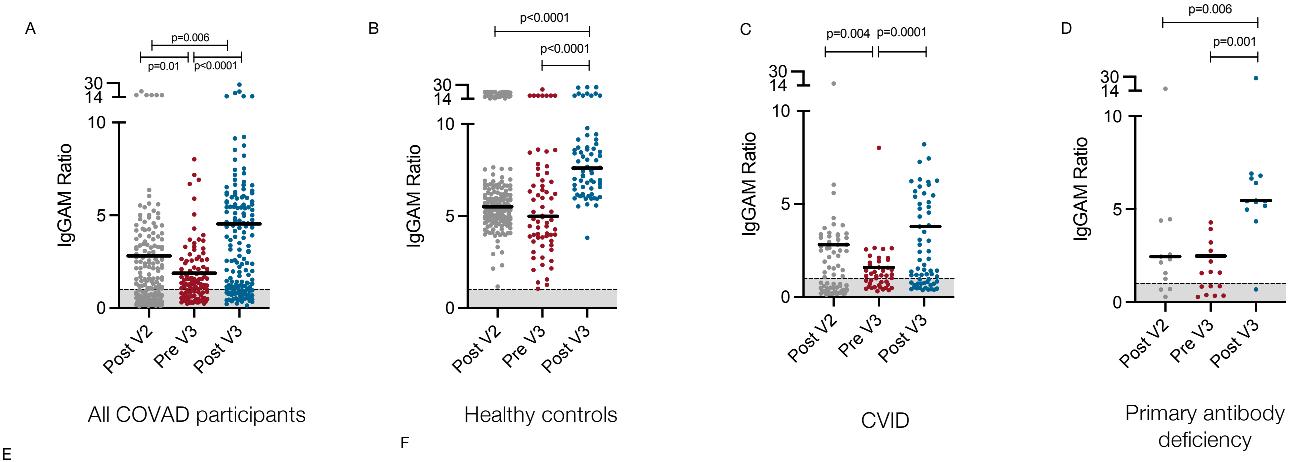


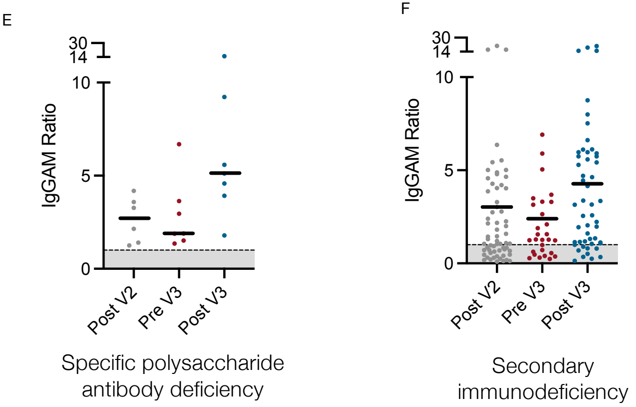


Supplementary Figure 1: Serological responses to third primary immunisation in healthy individuals and specific immunodeficiencies. Comparison of total anti-spike serological responses of COV-AD or control study participants 1-2 months post second vaccine dose (Post V2), up to 1 month prior to third vaccine dose (Pre V3) and 1-2 months post third vaccine dose (Post V3). (A) All COVAD participants. (B) Healthy controls. (C) Common variable immunodeficiency (D) Primary antibody deficiency (E) Specific polysaccharide antibody deficiency (F) Secondary immunodeficiency
